# Comparison of Artificial Intelligence-Based Applications for Mandible Segmentation: From Established Platforms to In-House-Developed Software

**DOI:** 10.3390/bioengineering10050604

**Published:** 2023-05-17

**Authors:** Robert R. Ileșan, Michel Beyer, Christoph Kunz, Florian M. Thieringer

**Affiliations:** 1 Department of Oral and Cranio-Maxillofacial Surgery, University Hospital Basel, 4031 Basel, Switzerland; michel.beyer@usb.ch (M.B.); christoph.kunz@usb.ch (C.K.); florian.thieringer@usb.ch (F.M.T.); 2 Medical Additive Manufacturing Research Group (Swiss MAM), Department of Biomedical Engineering, University of Basel, 4123 Allschwil, Switzerland

**Keywords:** artificial intelligence, mandible, segmentation, 3D virtual reconstruction, CBCT, CT, Convolutional Neural Networks, comparison, in-house, software, patch size, Cranio-Maxillofacial surgery, DICOM

## Abstract

Medical image segmentation, whether semi-automatically or manually, is labor-intensive, subjective, and needs specialized personnel. The fully automated segmentation process recently gained importance due to its better design and understanding of CNNs. Considering this, we decided to develop our in-house segmentation software and compare it to the systems of established companies, an inexperienced user, and an expert as ground truth. The companies included in the study have a cloud-based option that performs accurately in clinical routine (dice similarity coefficient of 0.912 to 0.949) with an average segmentation time ranging from 3′54″ to 85′54″. Our in-house model achieved an accuracy of 94.24% compared to the best-performing software and had the shortest mean segmentation time of 2′03″. During the study, developing in-house segmentation software gave us a glimpse into the strenuous work that companies face when offering clinically relevant solutions. All the problems encountered were discussed with the companies and solved, so both parties benefited from this experience. In doing so, we demonstrated that fully automated segmentation needs further research and collaboration between academics and the private sector to achieve full acceptance in clinical routines.

## 1. Introduction

The segmentation of anatomical structures is a process that virtually reconstructs the region of interest from medical images in three dimensions. It helps the physician prepare for surgical interventions and virtual surgical planning (VSP), visualize and interact with the patient’s anatomy (through three-dimensional (3D) printing or augmented and virtual reality (AR/VR)), and improve the medical outcome [1,2,3,4,5,6]. Until recently, the segmentation process was either manual, where the anatomical structure was labeled slice by slice, or semi-automatic, where the software identifies the region of interest and excludes other anatomical structures based on the selected threshold, marked points, or other user inputs [7,8,9,10]. Both segmentation types are subjective, time-intensive, and require specialized personnel. Artificial intelligence (AI)-based technologies are gradually being integrated into the clinical routine, and some companies already offer fully automated cloud-based solutions [11,12]. The most common techniques used for automatic segmentation are Statistical Shape Analysis [13] and Convolutional Neuronal Networks (CNNs) [14]. The last-mentioned technique has proven itself to be especially helpful for automatic segmentation [15,16,17]; for biomedical image segmentation, the U-Net architecture exhibits state-of-the-art performance [18]. In some cases, both techniques are combined to further improve segmentation accuracy [19]. Especially in the Cranio-Maxillofacial (CMF) field, due to the complex anatomy of the face, AI-based segmentation solutions could be advantageous and lead to fully automated virtual surgical planning workflows.

### Related Work

Previously conducted research has shown promising results for fully automated segmentation using different Convolutional Neural Network (CNN) architectures. Verhelst P.J. et al. [12] proposed a system for mandible segmentation in which two different 3D U-Net CNNs were trained in two phases with 160 cone-beam computed tomography (CBCT) images of the skull from orthognathic surgery patients. The automatically generated mandibles were compared to user-refined AI segmentations and semi-automatic ones, obtaining dice similarity coefficients of 0.946 and 0.944, respectively.

In a different approach, Lo Giudice A. et al. [20] proposed a fully convolutional deep encoder–decoder network that was trained on the MICCAI Head and Neck 2015 dataset and fine-tuned on 20 additional CBCT images. The segmentations were cut so that only the mandibular bone was considered for the assessment. The achieved dice similarity coefficient in comparison to the manual segmentations was 0.972. Apart from the mandibles, other anatomical structures of the skull were also automatically segmented with CNNs. One paper, which was published by Li Q. et al. [21], proposed a method that used a deep Convolutional Neural Network to segment and identify teeth from CBCT images. Another publication, from Kwak G.H. et al. [22], presented an automatic inferior alveolar canal detection system with different U-Net variants (3D SegNet, 2D U-Net, and 3D U-Net), where the three-dimensional U-Net performed best.

Deep learning technologies have improved in terms of performance and accuracy in recent years due to the growing accessibility of new technologies and global digitalization. This has encouraged the development of automatic diagnosis software in dentistry, as shown by Ezhov M. et al. [16], who evaluated a deep learning-based system to determine its real-time performance on CBCT images for five different applications (segmentation of jaw and teeth, tooth localization, numeration, periodontitis module, caries localization, and periapical lesion localization). The same researchers developed an AI-based evaluation tool for the pharyngeal airway in obstructive sleep apnea patients [17].

Other researchers, such as Yang W.F. et al. [11], used Mimics Viewer (Materialise) to segment the skull bones automatically. Compared to the ground truth, the segmented maxilla and mandible achieved dice similarity coefficient scores of 0.924 and 0.949, respectively. Although strenuous, Magnetic Resonance Imaging (MRI) segmentation of soft tissue has gained importance for VSP, as shown by Musatian S.A. et al. [23], who presented solutions for orbit and brain tumor segmentation based on CNNs. One software that is used in this study for semi-automatic segmentation is Brainlab IPlan.

Considering the gains of the last decade’s affordable computing power and a better understanding of AI programming, we decided to develop an automatic segmentation software and assess its performance in the clinical routine. The main research question was to determine how close non-professional medical personnel in the field of CMF/AI for automated segmentation applications could achieve the level of established companies (including the leading players and known start-ups). For that, we set up a research protocol that included the development of in-house segmentation software, followed by comparing an expert and an inexperienced user with a good anatomical understanding of the selected companies.

We use brand names that are/can be protected but are not marked with ^®^.

## 2. Materials and Methods

**Figure 1:**
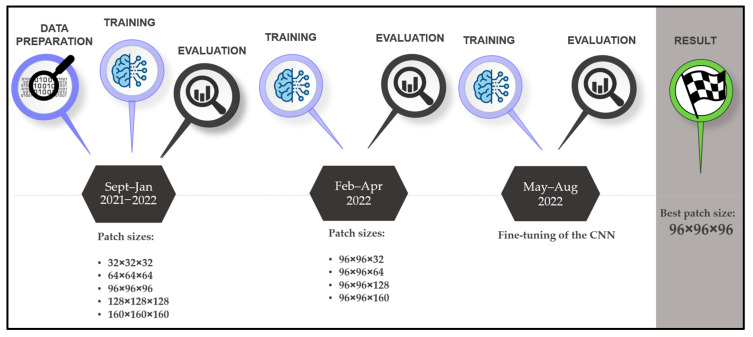
Timeline of the CNN development.

Our research protocol consists of setting up a fully automatic in-house segmentation software and comparing it with segmentation applications developed by established companies and manual segmentations performed by an inexperienced user with good anatomical understanding (surgeon with less than 50 segmentations) with regard to the ground truth performed by an expert (researcher with over 500 segmentations). We selected 210 head and neck DICOM (Digital Imaging and Communications in Medicine) files, where the mandibles were manually segmented. The comparison was made with twenty selected and anonymized DICOMs (ten computed tomography (CT) and ten cone-beam computed tomography (CBCT) images, with and without artifacts), where the expert provided the ground truth. For the analysis, we used standard surface- and volume-based metrics. For all segmentation steps, the time was measured (segmentation duration and postprocessing time: filling, smoothing, and exporting). The CNN development timeline is shown in Figure 1.

### 2.1. Statistical Analysis

The accuracy of the mandible segmentations was measured using the dice similarity coefficient (DSC), average surface distance (ASD), Hausdorff distance (HD), relative volume difference (RVD), volumetric overlap error (VOE), false positive rate (FPR), and false negative rate (FNR). The formulas for the calculation of these metrics are shown in Table 1.

### 2.2. CNN Development

#### 2.2.1. Training and Validation Data

For the training and validation of the Convolutional Neural Network (CNN), we relied on open-source data containing 504 DICOMs (Fluorodeoxyglucose-Positron Emission Tomography (FDG-PET) and CT images) of 298 patients that were diagnosed with cancer in the head and neck area. The databank is offered by the McGill University, Montreal, Canada, and the data acquisition took place between April 2006 and November 2014 [24]. A total of 160 DICOM files were selected to obtain heterogeneity regarding gender distribution, resolution, artifacts, and dentition, as shown in Table 2. The number of slices varies between 90 and 348, with an average of 170.5. The pixel spacing in the X and Y directions varies from 0.88 × 0.88 mm to 1.37 × 1.37 mm, whereas the slice thickness varies from 1.5 mm to 3.27 mm. The extended list is shown in Annex S1. The DICOM files were distributed among two datasets: the training dataset with 120 samples (60 with artifacts and 60 without artifacts) and the validation dataset with 40 samples (20 with artifacts and 20 without artifacts). Exclusion criteria were images of patients with brackets and osteosynthesis materials (screws and plates).

#### 2.2.2. Test Data

For the test dataset, 10 CT and 10 CBCT images from the University Hospital of Basel were selected. Both subgroups contained five DICOM files with metallic artifacts and five without. The number of slices ranges from 169 to 489, with a mean value of 378. The pixel spacing in X and Y directions ranges from 0.25 × 0.25 mm to 0.59 × 0.59 mm, with a mean value of 0.35 × 0.35 mm, and the slice thickness varies from 0.25 mm to 3.0 mm, with a mean value of 0.71 mm. None of the CT images have an isotropic voxel spacing (voxel spacing and slice thickness have the same value), whereas 9 out of 10 CBCTs have isotropic spacing. These images are representative of the ones used in the clinical routine; therefore, they differ greatly in aspects such as image dimension, voxel spacing, layer thickness, noise, etc. The same exclusion criteria were applied for the test dataset as for the training dataset. All images were anonymized.

#### 2.2.3. Segmentation

The DICOMs for the training and validation were imported into Mimics Innovation Suite (Version 24.0, Materialise NV, Leuven, Belgium), whereas the test samples were imported later into Mimics Innovation Suite Version 25.0. A semi-automatic segmentation workflow was applied using the Threshold, Split Mask, Region Grow, Edit Mask, Multiple Slice Edit, Smart Fill, and Smooth Mask tools. The teeth were included in the segmentation, and the masks were filled (i.e., they do not contain any voids). The mandible and the inferior nerve canal were labeled as a single mask and exported as a Standard Tessellation Language (STL) file.

#### 2.2.4. Model Architecture

**Figure 2:**
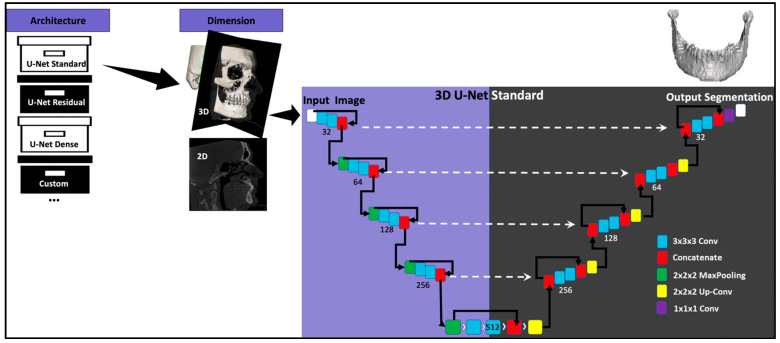
Architecture of the Convolutional Neural Network.

For the automatic segmentation of the mandible, the Medical Image Segmentation with Convolutional Neural Networks (MIScnn) Python library, Version 1.2.1 to 1.4.0 [25], was used. As architecture, a 3D U-Net, a Convolution Neural Network, was selected (Figure 2), which was developed for biomedical image segmentation [26]. The number of filters in the first layer (N filters) was set to 32, the number of layers of the U-Net structure (depth) was set to 4 as an activation function, the sigmoid function was used, and batch normalization was activated. The dice cross-entropy function was chosen as a loss function, which is a sum of the soft Dice Similarity Coefficient and the Cross-Entropy [27]. As normalization, the Z-score function was applied, and the image was resampled using a voxel spacing of 1.62 × 1.62 × 3.22 mm. The clipping subfunction was implemented to clip pixel values in a range between 50 and 3071 of the Hounsfield scale. The learning rate was set to 0.0001 at the beginning of the training, but through the Keras Callback function, it was reduced to 0.00001 once no further improvement was observed, with a patience of 10 epochs. Scaling, rotation, elastic deformation, mirroring, brightness, contrast changes, and Gaussian noise were used for data augmentation (a method to increase the number of training samples by slightly modifying/newly creating DICOMs from existing data to avoid overfitting and to improve the performance of the CNN). The models were trained for 1000 epochs with a NVIDIA RTX 3080 GPU (12 GB of VRAM), 64 GB of RAM, and an i9-11950H processor. The training time was about 100 h per model.

**Figure 3:**
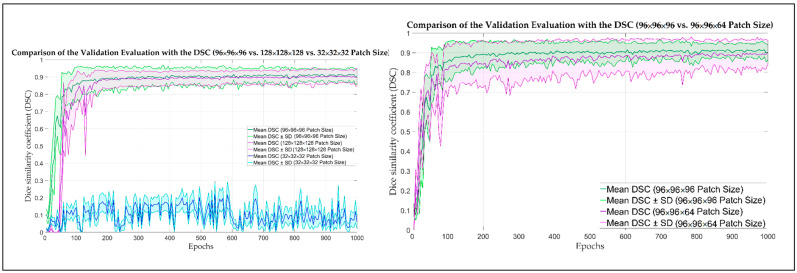
Graph of the evolution of the dice similarity coefficient (DCS) and its standard deviation (SD) of the validation samples for different patch size.

The CNN was trained in a two-phase approach. Firstly, the model was trained using five different cubical patch sizes (32 × 32 × 32, 64 × 64 × 64, 96 × 96 × 96, 128 × 128 × 128, and 160 × 160 × 160). In the second phase, the height of the best-performing input volume (96 × 96 × 96) was modified along the Z axis. Five further models with patch sizes of 96 × 96 × 32, 96 × 96 × 64, 96 × 96 × 128, and 96 × 96 × 160 were trained. The results are displayed in Table 3. The model trained with the 96 × 96 × 96 patch size (Figure 3) was the best-performing and, hence, was further improved by training it with 50 additional CT images from the University Hospital, Basel, and its performance was tested on the test dataset.

### 2.3. Software Comparison

#### 2.3.1. Relu

**Figure 4:**
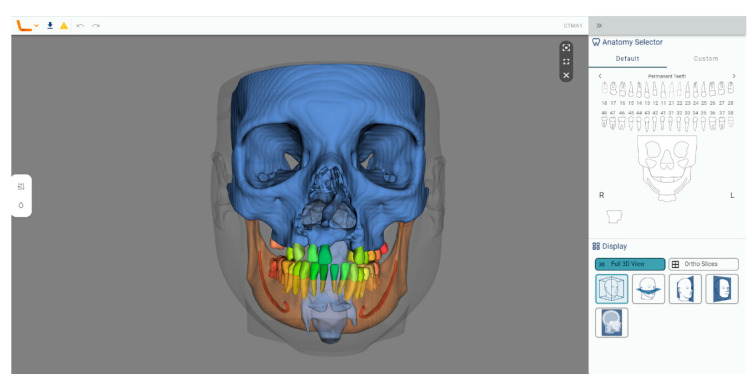
Relu’s user interface (CT w/A 1 displayed).

Relu (Figure 4) is an established start-up that offers fully automated cloud-based segmentation for CBCT and CT images for applications in the Cranio-Maxillofacial field. The segmented anatomical structures are the toothless mandible, the mandibular teeth (each tooth individually), the inferior alveolar canal, the toothless maxillary complex, the maxillary teeth (each tooth individually), the maxillary sinuses, the pharynx, and the soft tissue. The bone segmentations include cortical and cancellous structures. Relu is ISO 13485 compliant and has a CE mark pending.

For the segmentation of the mandible, the anonymized DICOM files of the test dataset were uploaded onto the cloud system (the company names it web application) and the segmentations were requested, but only for the mandible, mandibular teeth, and the inferior nerve canal, since these are the analyzed structures. After the segmentation was completed, these structures were combined directly in the cloud and downloaded as one STL file. This was then imported into Mimics (Version 25.0) and transformed into a mask, which was then manually filled with the “Smart Fill” tool. Afterward, the part was transformed into an object using the “Calculate Part tool”, smoothed for 4 iterations with the “Smooth” tool at a factor of 0.4, and finally exported as an STL file.

With Relu, we encountered problems in 3 of the 20 test DICOMs during the segmentation process regarding voxel spacing, image orientation, and cropping. All transmitted problems were solved by the support team.

#### 2.3.2. Materialise Mimics Viewer

**Figure 5:**
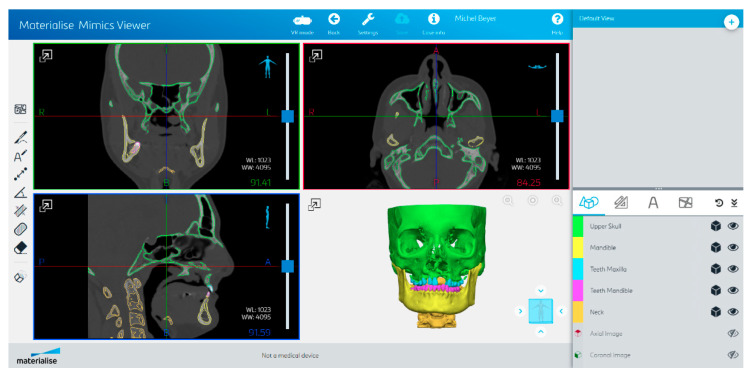
Materialise Viewer’s user interface (CT w/A 1 displayed).

The Materialise Viewer (Figure 5) is a cloud-based platform for online visualization and segmentation of DICOM files. Fully automatic segmentation can be requested for CMF CBCT, heart CT, shoulder CT, hip CT, knee CT, knee MRI, and all bones CT. The Mimics Automatic Algorithms are part of the FDA 510(k) of Mimics Medical and standalone CE-marked medical devices.

For the segmentation of the mandible, the CMF CBCT segmentation algorithm was used, which was designed to segment both CBCT and CT. The anonymized DICOM files of the test dataset were inserted into a Mimics file, which was then uploaded onto Mimics Viewer and the segmentation was requested. The output of the fully automatic segmentation was a Mimics file containing five segmented parts, which are called “Upper skull”, “Mandible”, “Teeth Maxilla”, “Teeth Mandible”, and “Neck”, containing the anatomy of skull and maxilla, mandible, maxillary teeth, mandibular teeth, and neck, respectively. Only the cortical bone was segmented in the Materialise Mimics Viewer, not the cancellous bone. The inferior alveolar canal was not segmented.

The file was opened with Mimics (Version 25.0) and the parts were transformed into masks using the “Mask from Object” tool. The mask containing the mandible and the one containing the mandibular teeth were combined, and the holes inside the mandible were filled manually with the “Smart Fill” tool in order to make volumetric comparisons possible. In the cases where there were some holes in the surface of the model, we filled them without intervening in the segmentation of the cortical bone. Afterward, the part was transformed into an object using the “Calculate Part tool”, smoothed for 4 iterations with the “Smooth” tool at a factor of 0.4, and finally exported as an STL file.

With Mimics Viewer, we encountered problems in 2 of the 20 test DICOMs during the segmentation process regarding image orientation and cropping. All transmitted problems were solved by the support team.

#### 2.3.3. Diagnocat

**Figure 6:**
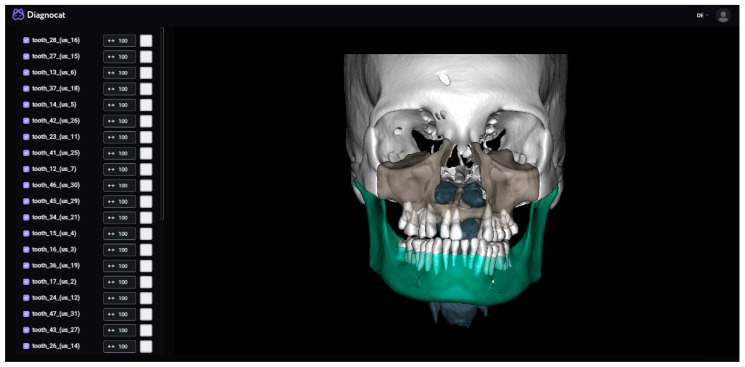
Diagnocat’s user interface (CT w/A 1 displayed).

Diagnocat (Figure 6) is an established start-up that offers fully automated segmentation for CBCT images and prediagnosis for 2D dental X-rays. The segmented anatomical structures are the toothless mandible, the mandibular teeth (each tooth individually), the inferior alveolar canal, the toothless maxilla, the maxillary teeth (each tooth individually), the cranium, the airways, and the soft tissue. The bone segmentations include cortical and cancellous structures. Diagnocat has a CE mark.

For the segmentation of the mandible, the anonymized DICOM files were uploaded onto the cloud system and the segmentations requested (all the structures as separated files option). After the segmentation was completed, the mandible, the inferior alveolar canal, and the mandibular teeth were downloaded and combined into a single file using Materialise 3-Matic (Version 17.0, Materialise NV, Leuven, Belgium). This was then imported into Mimics (Version 25.0) and transformed into an object using the “Calculate Part tool”, smoothed for 4 iterations with the “Smooth” tool at a factor of 0.4, and finally exported as an STL file.

With Diagnocat, we encountered problems in all of the CT images and one CBCT image out of the twenty test DICOMs during the segmentation process. All these images had non-isotropic voxel spacing (CBCTs generally have isotropic voxel spacing, as shown in Annex S1–S5), which needed to be adapted. All transmitted problems were solved by the support team.

#### 2.3.4. Brainlab

**Figure 7:**
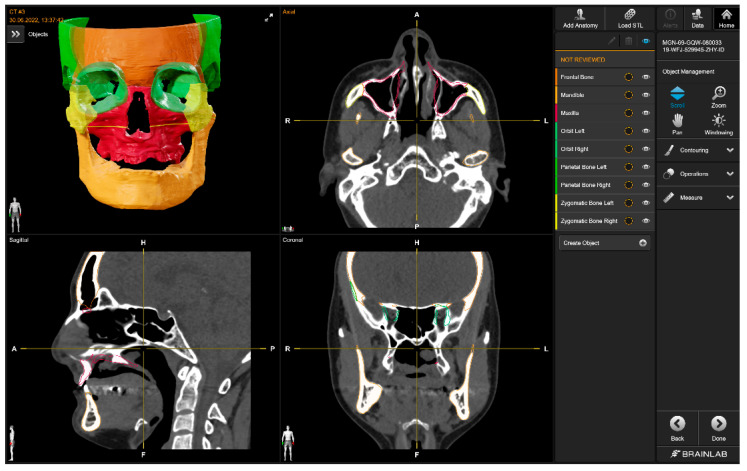
Brainlab’s user interface (CT w/A 1 displayed).

The Brainlab Elements application (Figure 7) consists of multiple applications and backend services for image processing of medical data (data transfer and exchange, image co-registration, automatic image segmentation, manual contouring, object manipulation, trajectory planning, etc.). The anatomical structures that can be automatically segmented are the optic nerve, eye, midface, skull base, skull base anterior, skull base central, skull base posterior, orbit volume, skull, ethmoid bone, LeFort I Template, LeFort II Template, LeFort III Template, LeFort III-I Template, mandible, mandible body, mandible ramus, frontal bone, maxilla, nasal bone, orbit, orbit floor, orbit wall medial, zygomatic bone, occipital bone, parietal bone, sphenoid bone, and temporal bone. For all bony structures, the cortical and cancellous bones are segmented by Brainlab. Teeth are not part of the segmentation model.

The mandible was downloaded as an STL file and was then imported into Mimics (Version 25.0) and transformed into a mask, which was then manually filled with the “Smart Fill” tool. Afterward, the part was transformed into an object using the “Calculate Part tool”, smoothed for 4 iterations with the “Smooth” tool at a factor of 0.4, and finally exported as an STL file.

With Brainlab, no problems were encountered during the segmentation process.

### 2.4. Mandible Cutting

**Figure 8:**
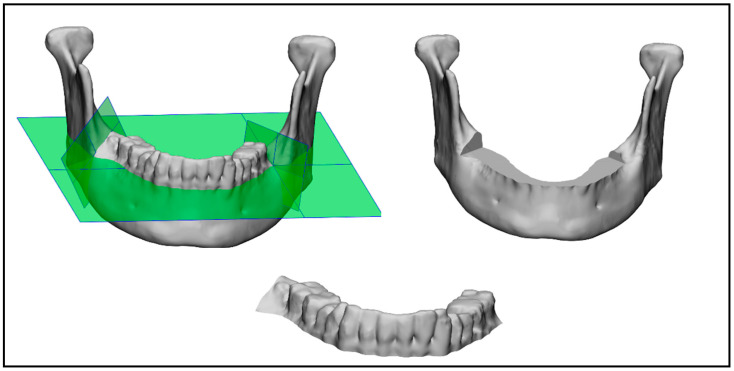
Cutting planes on mandible with teeth (**left**), mandibular bone (**right**), and mandibular teeth (**bottom**).

The following three comparisons were made: one of the mandible with teeth, one of just the mandibular bone, and the last of just the mandibular teeth (as shown in Figure 8). In order to split the mandible into the mandibular teeth and the mandibular bone, 3-Matic was used. For each of the 20 mandibles in the test dataset, the ground truth was used to manually insert three cutting planes (one horizontal and two vertical planes), which were used to automatically cut and split the segmented mandibles for each company using the 3-Matic scripting tool. Two different STL files were obtained, one containing the mandibular bone and one containing the mandibular teeth.

## 3. Results

The main results after all the assessments were made are as follows:

- Overall, Relu performed best if the mean DSC for the mandible with teeth (mean DSC of 0.938) and bone (mean DSC of 0.949) is taken into consideration, which was closely followed by Diagnocat and then Materialise, as displayed in Table 4 and Table 5.
- Brainlab was only included for the assessment of the mandibular bone, as it does not offer teeth segmentation (mean DSC of 0.912), as displayed in Table 5.
- Materialise performed best over the other software in the assessment of the mandibular teeth (mean DSC of 0.864), as displayed in Table 6.
- We could observe that in all assessments, our in-house-developed software performed worst, obtaining the closest result in the mandibular bone comparison (mean DSC of 0.894), but achieved an accuracy of 94.24% in comparison to the best-performing software, as displayed in Table 4, Table 5 and Table 6.
- The segmentation performed by the inexperienced user with good anatomical understanding (CMF surgeon) had, for all assessments, the best mean DSC, as displayed in Table 4, Table 5 and Table 6.

**Figure 9:**
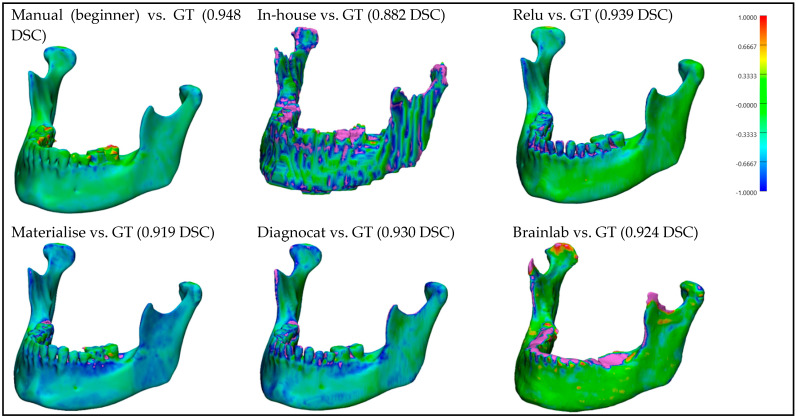
**CT with artifacts:** Color mapping of the surface distance between the segmented mandibles of the CT w/A 1 image and the ground truth (GT).

**Figure 10:**
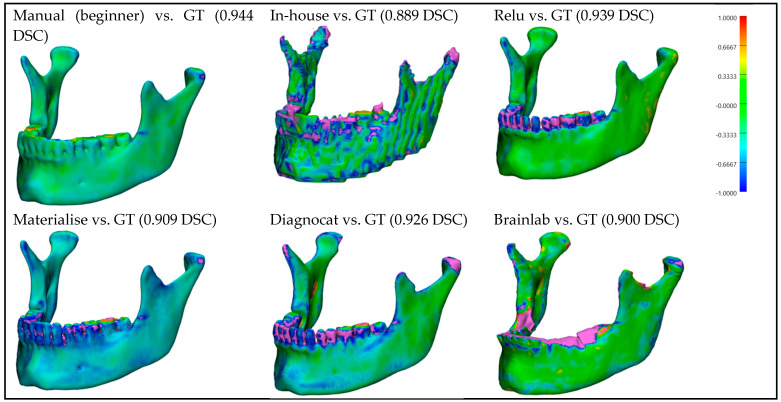
**CT without artifacts:** Color mapping of the surface distance between the segmented mandibles of the CT w/o A 1 image and the ground truth (GT).

**Figure 11:**
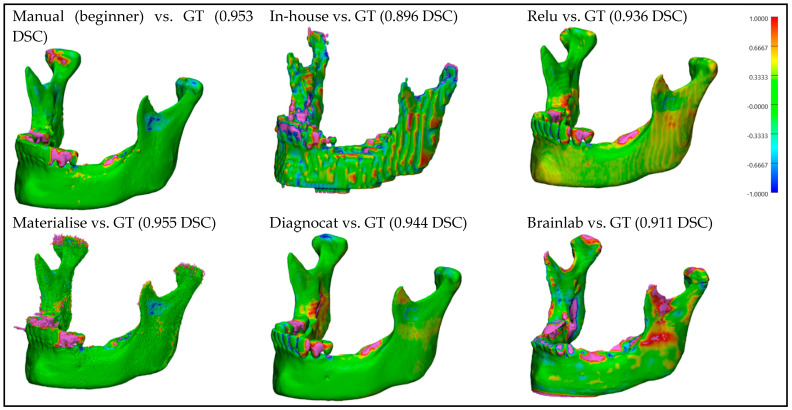
**CBCT with artifacts:** Color mapping of the surface distance between the segmented mandibles of the CBCT w/A 1 image and the ground truth (GT).

**Figure 12:**
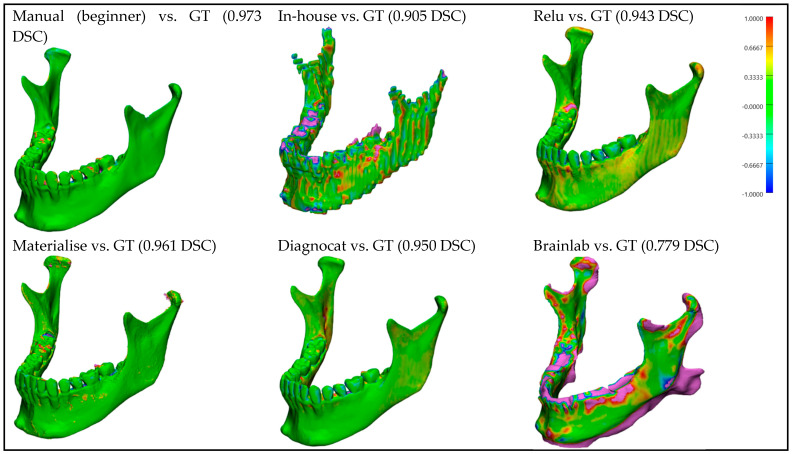
**CBCT without artifacts:** Color mapping of the surface distance between the segmented mandibles of the CBCT w/o A 1 image and the ground truth (GT).

For better visualization and understanding of the results, we chose to display in each category (CT with artifacts (Figure 9), CT without artifacts (Figure 10), CBCT with artifacts (Figure 11), and CBCT without artifacts (Figure 12)) the first segmented mandible. For that, we used the color mapping of the surface distance between the segmentation and the ground truth (where the segmentation is visible and the ground truth is hidden), with minimum and maximum ranges of −1.0 mm and +1.0 mm.

**Figure 13:**
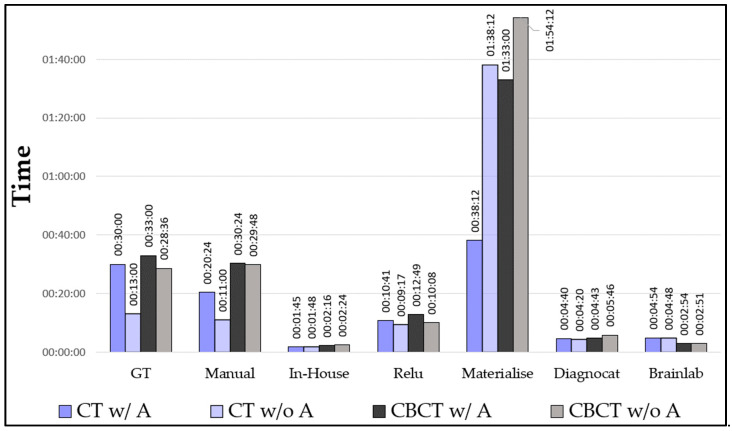
Graph of the mean timing for the segmentations.

Timing: We calculated the mean values of the segmentation times for CT and CBCT with/without artifacts (Figure 13). We have shown that our in-house model performed best with the lowest mean time (2′03″), followed by Brainlab (3′54″) and Diagnocat (4′52″). The manually segmented mandibles (those from the expert and the inexperienced user) showed similar timings (26′09″ and 22′54″, respectively). Materialise showed the highest mean value (85′54″).

## 4. Discussions

In a clinical routine, three important factors stand out: segmentation accuracy, cost, and time. The segmentation accuracy result was best for manual segmentation in all comparisons, followed by Relu, Diagnocat, and Materialise, which all performed very similarly to one another. Brainlab could only be included in the comparison of the mandibular bone because the segmentation did not include the teeth, as its main activity offers intraoperative navigation solutions. Our in-house-developed CNN performed worst in all of the comparisons. We encountered the problem that the segmented mandibles of our in-house CNN had a cubical surface, which was probably due to a too high voxel spacing parameter. This problem could not be fixed and will require further training and improvements to the model. The advantage of our system is that it has higher stability than the other software included in our study. We could upload all the DICOM files without any modifications and obtain a complete segmentation. The other software encountered some problems with DICOMs containing not only the skull but also, e.g., the thorax, and needed preprocessing (cropping) in order to obtain the segmentation. A further problem was with the handling of CT images, because some systems were only trained on CBCT images, and in many cases, images without isotropic voxel spacing were not supported and had to be modified. Additionally, it is worth mentioning that not all the DICOM file orientations were supported. Figure 9 and Figure 10 show that for CT images, the segmented mandibles from Materialise and Diagnocat had a slight inaccuracy in the segmentation of the mandibular bone compared to those from Relu or Brainlab, which was probably due to different thresholds used for the clipping during the training. Finally, the manual segmentation may have performed better than other automatic systems due to a similar segmentation protocol as the one for the ground truth. The same could apply to our in-house-developed CNN, which may have performed better because it was trained with a dataset prepared by following the same segmentation protocol. Using Mimics, which is developed by Materialise, for the manual segmentation (training and test data) and the filling process, could have had a positive influence on the final outcomes. Furthermore, the filling process of the mandibles, which was performed manually and was needed due to the different segmentation approaches, could be subject to bias. Pricing is also a relevant factor that needs to be considered. As we were offered the segmentations by the companies for research purposes, pricing was not further investigated in this study. The timing may vary due to the fact that most of the companies offer a cloud service, which, depending on the server load and internet connection, affects the segmentation time. Additionally, our ground truth implies that a manual segmentation process can differ from the anatomical specimen ground truth, which implies a scanning process. Other studies are necessary to compare the segmentations with laser-scanned mandibles (anatomical specimens) as the ground truth to improve accuracy.

## 5. Conclusions

In our study, we wanted to find out if non-professional medical personnel could become close to segmentation software developed by established companies, following a clearly defined research protocol. The results showed that our in-house-developed model achieved an accuracy of 94.24% compared to the best-performing software. We also conclude that the segmentation performed by an inexperienced user with good anatomical understanding achieved the best result compared to all the other companies included in the study.

The timing required to automatically segment a mandible was, for almost all of the software, lower than the manual segmentation.

We can deduce that in order to obtain better quality segmentations, the CNN has to be trained with a dataset containing a large number of highly variable images (e.g., older and newer DICOM files, different types of DICOMs (CT and CBCT), and different image sizes, including different regions of interest and from different centers) that is constantly updated and enlarged due to the constantly improving image technologies.

To fulfill today’s expectations of personalized medicine, digital workflows, including segmentation, need to offer stable solutions. Answers must be found for the current problems that are often encountered during the segmentation process: artifacts, amount of noise, voxel spacing, the size of the image, DICOM type, and image orientation. All these problems were reported to the companies so that solutions could be elaborated in the future.

For the future, the first step for implementing fully automated digital workflows is to generate accurate segmentations of the patient’s anatomy, which will be possible after solving the above-mentioned issues.

Once the above-mentioned issues are solved, these software can be implemented in fully automated digital workflows, allowing new clinical applications, such as intraoperatively 3D-printed patient-specific implants, even in emergency situations.

## Figures and Tables

**Table 1:** List of the metrics used in this study and their formula.

**Metric**	**Formula**	**Legend**
Dice similarity coefficient (DSC)	$DSC=\frac{2\left|A\cap B\right|}{\left|A\right|+\left|B\right|}=\frac{2\mathrm{TP}}{2TP+FP+FN}$	The dice similarity coefficient measures the similarity between two sets of data.
Average surface distance (ASD)	$ASD=\frac{1}{n_{A}+n_{B}}\left(\sum_{i=1}^{n_{A}}\underset{b\in B}{\min}{||a_{i}-b||}_{2}+\sum_{j=1}^{n_{B}}\underset{a\in A}{\min}{||b_{j}-a||}_{2}\right)$	The average surface distance is the average of all the distances between the surfaces of the ground truth and the volume.
Hausdorff distance (HD)	$\underset{}{d_{H}=\max}\{\sup_{x\in X}d\left(x,Y\right),\sup_{y\in Y}d\left(X,y\right)\}$	The Hausdorff distance is the maximum distance between the ground truth and the volume.
Relative volume difference (RVD)	$RVD=\frac{\left|B\right|-\left|A\right|}{\left|A\right|}$	The relative volume difference measures the absolute size difference of the ground truth to the volume as a fraction of the ground truth.
Volumetric overlap error (VOE)	$VOE=1-\frac{DSC}{2-DSC}$	The volumetric overlap error is the corresponding error metric of the dice similarity coefficient.
False positive rate (FPR)	$FPR=\frac{FP}{FP+TN}$	The false positive rate is the probability that a positive result is given when the true value is negative.
False negative rate (FNR)	$FNR=\frac{FN}{FN+TP}$	The false negative rate or miss rate is the probability that the analysis misses a true positive.

**Table 2:** List of characteristics of the images used for the training of the Convolutional Neural Network.

Nr. Studies	With Artifacts	Without Artifacts—With Teeth	Without Artifacts—Without Teeth (Edentulous)
Female	33	12	19
Male	47	28	21
Male and Female	80	40	40

**Table 3:** The patch sizes with which the CNNs were trained; the reached dice similarity coefficient (DSC) and its standard deviation (SD); and the epoch when it was reached.

Patch Size	Max. DSC	SD	Epoch
32×32 × 32	0.222	0.073	545
64 × 64 × 64	0.838	0.110	840
96 × 96 × 32	0.857	0.067	635
96 × 96 × 64	0.902	0.060	1000
**96 × 96 × 96**	**0.916**	**0.033**	**975**
96 × 96 × 128	0.878	0.087	995
96 × 96 × 160	0.852	0.147	810
128 × 128 × 128	0.907	0.038	915
160 × 160 × 160	0.860	0.077	725

**Table 4:** Mean dice similarity coefficient (DSC) of the **mandible with teeth** comparison.

	Manual (Beginner)	In-House	Relu	Materialise	Diagnocat	Brainlab
Mean CT w/A	0.961	0.885	0.939	0.914	0.927	-
Mean CT w/o A	0.968	0.891	0.935	0.903	0.921	-
Mean CBCT w/A	0.951	0.863	0.938	0.947	0.941	-
Mean CBCT w/o A	0.958	0.899	0.939	0.956	0.947	-
Mean	**0.960**	**0.884**	**0.938**	**0.930**	**0.934**	-

**Table 5:** Mean dice similarity coefficient (DSC) of the **mandibular bone** comparison.

	Manual (Beginner)	In-House	Relu	Materialise	Diagnocat	Brainlab
Mean CT w/A	0.968	0.898	0.958	0.925	0.943	0.948
Mean CT w/o A	0.969	0.900	0.952	0.909	0.936	0.943
Mean CBCT w/A	0.963	0.873	0.944	0.959	0.948	0.852
Mean CBCT w/o A	0.962	0.905	0.943	0.958	0.950	0.903
Mean	**0.966**	**0.894**	**0.949**	**0.938**	**0.944**	**0.912**

**Table 6:** Mean dice similarity coefficient (DSC) of the **mandibular teeth** comparison.

	Manual (Beginner)	In-House	Relu	Materialise	Diagnocat	Brainlab
Mean CT w/A	0.923	0.787	0.814	0.838	0.817	-
Mean CT w/o A	0.953	0.818	0.792	0.847	0.797	-
Mean CBCT w/A	0.838	0.762	0.858	0.837	0.853	-
Mean CBCT w/o A	0.935	0.841	0.889	0.935	0.903	-
Mean	**0.912**	**0.802**	**0.838**	**0.864**	**0.842**	-

## Data Availability

Data are contained within the article. Additional information can be obtained from the corresponding author upon reasonable request.

## Supplementary Materials

The following supporting information can be downloaded at: https://www.mdpi.com/article/10.3390/bioengineering10050604/s1, Annex S1: Test data DICOM properties; Annex S2: Dice similarity coefficient (DSC) of the mandible with teeth comparison; Annex S3: Dice similarity coefficient (DSC) of the mandibular bone comparison; Annex S4: Dice similarity coefficient (DSC) of the mandibular teeth comparison; Annex S5: Mean values for the comparison of the mandible with teeth segmentations, mandibular bone and mandibular teeth to the ground truth by using the dice similarity coefficient (DSC), average surface distance (ASD), Hausdorff distance (HD), relative volume difference (RVD), volumetric overlap error (VOE), false positive rate (FPR), and false negative rate (FNR).

## Abbreviations

3D	Three-dimensional
AI	Artificial Intelligence
AR	Augmented Reality
ASD	Average Surface Distance
CBCT	Cone-Beam Computed Tomography
CMF	Cranio-Maxillofacial
CNN	Convolutional Neural Network
CT	Computed Tomography
DICOM	Digital Imaging and Communications in Medicine
DSC	Dice Similarity Coefficient
FDG-PET	Fluorodeoxyglucose-Positron Emission Tomography
GT	Ground Truth
FNR	False Negative Rate
FPR	False Positive Rate
HD	Hausdorff distance
MIScnn	Medical Image Segmentation with Convolutional Neural Networks
RAS	Right, Anterior Superior
RVD	Relative Volume Difference
SD	Standard Deviation
STL	Standard Tessellation Language
VOE	Volumetric Overlap Error
VR	Virtual Reality
VSP	Virtual Surgical Planning
