# Supplementary material for: Comparison of Artificial Intelligence-Based Applications for Mandible Segmentation: From Established Platforms to In-House-Developed Software
Source: Bioengineering (Basel). 2023 May 17;10(5):604. doi: 10.3390/bioengineering10050604 (PMC10215609; doi:10.3390/bioengineering10050604)
Supplement: Supplementary file 1 [file bioengineering-10-00604-s001.zip › bioengineering-2392515-supplementary.pdf]

Annexes

Annex S1 Test data DICOM properties

	Voxel spacing (X, Y)	Slice thickness	Nr. Slices	Orientation
**CT w/ A 1**	0.357	1.0	337	Axial
**CT w/ A 2**	0.586	0.75	440	Axial
**CT w/ A 3**	0.344	0.75	317	Axial
**CT w/ A 4**	0.475	3.0	213	Axial
**CT w/ A 5**	0.334	0.75	325	Axial
**CT w/o A 1**	0.479	1.0	341	Axial
**CT w/o A 2**	0.490	0.75	331	Axial
**CT w/o A 3**	0.449	1.0	293	Axial
**CT w/o A 4**	0.502	1.0	483	Axial
**CT w/o A 5**	0.441	1.0	489	Axial
**CBCT w/ A 1**	0.25	0.25	421	Axial
**CBCT w/ A 2**	0.25	0.25	424	Axial
**CBCT w/ A 3**	0.25	0.25	424	Axial
**CBCT w/ A 4**	0.25	0.25	424	Axial
**CBCT w/ A 5**	0.327	1.0	169	Sagittal
**CBCT w/o A 1**	0.25	0.25	424	Axial
**CBCT w/o A 2**	0.25	0.25	421	Axial
**CBCT w/o A 3**	0.25	0.25	424	Axial
**CBCT w/o A 4**	0.25	0.25	421	Axial
**CBCT w/o A 5**	0.3	0.3	443	Axial
Mean	*0.3542*	*0.715*	*378.2*	-

Annex S2 Dice similarity coefficient (DSC) of the mandible with teeth comparison

	Manual (beginner)	In-house	Relu	Materialise	Diagnocat	Brainlab
CT w/ A 1	0.948	0.882	0.939	0.919	0.930	0.924
CT w/ A 2	0.967	0.906	0.949	0.932	0.943	0.910
CT w/ A 3	0.979	0.898	0.954	0.941	0.947	0.917
CT w/ A 4	0.932	0.862	0.919	0.863	0.888	0.883
CT w/ A 5	0.980	0.877	0.932	0.918	0.927	0.915
Mean CT w/ A	**0.961**	**0.885**	**0.939**	**0.914**	**0.927**	**0.910**
CT w/o A 1	0.944	0.889	0.939	0.909	0.926	0.900
CT w/o A 2	0.964	0.886	0.934	0.895	0.914	0.897
CT w/o A 3	0.984	0.912	0.954	0.915	0.938	0.922
CT w/o A 4	0.974	0.890	0.914	0.898	0.916	0.909
CT w/o A 5	0.973	0.878	0.932	0.896	0.913	0.886
Mean CT w/o A	**0.968**	**0.891**	**0.935**	**0.903**	**0.921**	**0.903**
CBCT w/ A 1	0.953	0.896	0.936	0.955	0.944	0.911
CBCT w/ A 2	0.944	0.885	0.932	0.930	0.942	0.828
CBCT w/ A 3	0.952	0.739	0.943	0.954	0.928	0.768
CBCT w/ A 4	0.958	0.893	0.944	0.959	0.948	0.878
CBCT w/ A 5	0.950	0.900	0.932	0.939	0.944	0.722
Mean CBCT w/ A	**0.951**	**0.863**	**0.938**	**0.947**	**0.941**	**0.821**
CBCT w/o A 1	0.973	0.905	0.943	0.961	0.950	0.779
CBCT w/o A 2	0.956	0.889	0.935	0.953	0.949	0.889

**CBCT w/o A 3**	0.954	0.897	0.940	0.955	0.949	0.861
**CBCT w/o A 4**	0.959	0.905	0.940	0.967	0.950	0.891
**CBCT w/o A 5**	0.947	0.901	0.938	0.942	0.938	0.912
Mean CBCT w/o A	**0.958**	**0.899**	**0.939**	**0.956**	**0.947**	**0.866**
Mean	0.960	0.884	0.938	0.930	0.934	0.875

Annex S3 Dice similarity coefficient (DSC) of the mandibular bone comparison

	**Manual (beginner)**	**In-house**	**Relu**	**Materialise**	**Diagnocat**	**Brainlab**
**CT w/ A 1**	0.949	0.891	0.951	0.923	0.937	0.960
**CT w/ A 2**	0.971	0.928	0.966	0.945	0.955	0.945
**CT w/ A 3**	0.980	0.904	0.969	0.946	0.958	0.963
**CT w/ A 4**	0.960	0.883	0.958	0.891	0.925	0.918
**CT w/ A 5**	0.981	0.884	0.947	0.923	0.940	0.955
Mean CT w/ A	**0.968**	**0.898**	**0.958**	**0.925**	**0.943**	**0.948**
**CT w/o A 1**	0.945	0.898	0.956	0.916	0.939	0.941
**CT w/o A 2**	0.965	0.894	0.952	0.901	0.930	0.937
**CT w/o A 3**	0.986	0.919	0.968	0.921	0.948	0.957
**CT w/o A 4**	0.977	0.900	0.932	0.904	0.933	0.951
**CT w/o A 5**	0.972	0.887	0.950	0.902	0.929	0.931
Mean CT w/o A	**0.969**	**0.900**	**0.952**	**0.909**	**0.936**	**0.943**
**CBCT w/ A 1**	0.969	0.910	0.945	0.970	0.953	0.936
**CBCT w/ A 2**	0.960	0.900	0.945	0.948	0.951	0.868
**CBCT w/ A 3**	0.955	0.738	0.945	0.958	0.933	0.774
**CBCT w/ A 4**	0.969	0.910	0.950	0.968	0.956	0.926
**CBCT w/ A 5**	0.9623	0.907	0.938	0.951	0.949	0.757
Mean CBCT w/ A	**0.963**	**0.873**	**0.944**	**0.959**	**0.948**	**0.852**
**CBCT w/o A 1**	0.976	0.916	0.944	0.961	0.951	0.825
**CBCT w/o A 2**	0.961	0.898	0.938	0.959	0.950	0.917
**CBCT w/o A 3**	0.961	0.899	0.942	0.954	0.950	0.911
**CBCT w/o A 4**	0.960	0.909	0.943	0.969	0.954	0.930
**CBCT w/o A 5**	0.952	0.904	0.950	0.948	0.946	0.933
Mean CBCT w/o A	**0.962**	**0.905**	**0.943**	**0.958**	**0.950**	**0.903**
Mean	0.966	0.894	0.949	0.938	0.944	0.912

Annex S4 Dice similarity coefficient (DSC) of the mandibular teeth comparison

	**Manual (beginner)**	**In-house**	**Relu**	**Materialise**	**Diagnocat**	**Brainlab**
**CT w/ A 1A**	0.931	0.810	0.848	0.871	0.860	0.584
**CT w/ A 2**	0.944	0.710	0.825	0.833	0.849	0.566
**CT w/ A 3**	0.974	0.838	0.851	0.889	0.852	0.423
**CT w/ A 4**	0.795	0.750	0.720	0.717	0.692	0.685
**CT w/ A 5**	0.972	0.827	0.827	0.879	0.830	0.552
Mean CT w/ A	**0.923**	**0.787**	**0.814**	**0.838**	**0.817**	**0.562**
**CT w/o A 1**	0.933	0.818	0.783	0.841	0.796	0.411
**CT w/o A 2**	0.948	0.823	0.790	0.841	0.790	0.536
**CT w/o A 3**	0.959	0.826	0.809	0.850	0.817	0.441
**CT w/o A 4**	0.968	0.827	0.802	0.865	0.807	0.569
**CT w/o A 5**	0.955	0.795	0.775	0.836	0.773	0.382
Mean CT w/o A	**0.953**	**0.818**	**0.792**	**0.847**	**0.797**	**0.468**
**CBCT w/ A 1**	0.789	0.725	0.826	0.800	0.841	0.509
**CBCT w/ A 2**	0.756	0.712	0.761	0.749	0.810	0.495
**CBCT w/ A 3**	0.850	0.729	0.885	0.853	0.789	0.583
**CBCT w/ A 4**	0.912	0.797	0.911	0.911	0.913	0.645
**CBCT w/ A 5**	0.885	0.847	0.907	0.875	0.912	0.352

Mean CBCT w/ A	**0.838**	**0.762**	**0.858**	**0.837**	**0.853**	**0.517**
**CBCT w/o A 1**	0.953	0.815	0.933	0.959	0.942	0.199
**CBCT w/o A 2**	0.936	0.804	0.913	0.927	0.932	0.561
**CBCT w/o A 3**	0.947	0.870	0.924	0.950	0.940	0.041
**CBCT w/o A 4**	0.957	0.869	0.921	0.961	0.927	0.547
**CBCT w/o A 5**	0.882	0.848	0.756	0.876	0.775	0.575
Mean CBCT w/o A	**0.935**	**0.841**	**0.889**	**0.935**	**0.903**	**0.384**
Mean	0.912	0.802	0.838	0.864	0.842	0.483

Annex S5 Mean values for the comparison of the mandible with teeth segmentations, mandibular bone and mandibular teeth to the ground truth by using the dice similarity coefficient (DSC), average surface distance (ASD), Hausdorff distance (HD), relative volume difference (RVD), volumetric overlap error (VOE), false positive rate (FPR) and false negative rate (FNR)

	**Mean DSC±SD**	**Mean ASD±SD**	**Mean HD±SD**	**Mean RVD±SD**	**Mean VOE±SD**	**Mean FPR±SD**	**Mean FNR±SD**
Manual (beginner)	0.960±0.0138	0.518±0.232	12.682±6.768	-0.00174±0.0418	0.077±0.025	0.000509±0.000423	0.0412±0.0261
	0.966±0.011	0.430±0.195	9.316±4.792	-0.00664±0.0389	0.066±0.0202	0.000362±0.000295	0.0375±0.0256
	0.912±0.64	0.672±0.586	16.389±24.734	0.0529±0.123	0.155±0.102	0.0001439±0.000165	0.066±0.046
In-house	0.884±0.035	1.609±0.641	37.393±67.022	-0.144±0.074	0.206±0.051	0.000489±0.000496	0.178±0.058
	0.894±0.038	1.476±0.668	37.121±67.109	-0.137±0.072	0.190±0.055	0.000376±0.000374	0.1660±0.0607
	0.802±0.049	1.554±0.541	16.056±7.056	-0.185±0.124	0.328±0.0667	0.000124±0.000132	0.272±0.065
Relu	0.938±0.0096	0.804±0.214	13.921±6.641	-0.00314±0.089	0.117±0.0170	0.000846±0.000863	0.0638±0.0438
	0.949±0.010	0.654±0.257	10.081±4.932	0.017±0.080	0.096±0.018	0.000757±0.000776	0.0427±0.0346
	0.838±0.063	1.131±0.401	12.584±6.974	-0.147±0.175	0.273±0.094	0.0000915±0.000113	0.221±0.112
Materialise	0.930±0.027	0.904±0.381	17.248±16.134	-0.061±0.103	0.130±0.047	0.000524±0.000595	0.0973±0.0714
	0.938±0.025	0.773±0.322	13.814±16.167	-0.057±0.096	0.116±0.043	0.000407±0.000458	0.0876±0.0674
	0.864±0.061	1.115±0.770	13.864±10.197	-0.073±0.212	0.234±0.094	0.000118±0.000178	0.168±0.101
Diagnocat	0.934±0.016	0.825±0.152	13.526±6.256	-0.037±0.090	0.123 ±0.0277	0.000652±0.000672	0.082±0.054
	0.944±0.0099	0.692±0.158	11.494±5.975	-0.0186±0.0832	0.105±0.018	0.000583±0.000591	0.064±0.046
	0.842±0.066	1.143±0.381	12.383±7.346	-0.174±0.149	0.267±0.099	0.0000736±0.0000901	0.228±0.113
Brainlab	0.875±0.055	2.194±1.465	34.265±21.534	-0.0095±0.1394	0.218±0.082	0.001577±0.001574	0.132±0.047
	0.912±0.058	1.454±1.309	27.811±23.439	0.0540±0.1285	0.157±0.091	0.001385±0.001419	0.067±0.036
	0.483±0.149	7.339±8.956	34.850±21.756	-0.4482±0.445	0.670±0.117	0.0002022±0.0003738	0.609±0.180
